# The Potential Role of Spermine and Its Acetylated Derivative in Human Malignancies

**DOI:** 10.3390/ijms23031258

**Published:** 2022-01-23

**Authors:** Ryan Tsz-Hei Tse, Christine Yim-Ping Wong, Peter Ka-Fung Chiu, Chi-Fai Ng

**Affiliations:** S.H. Ho Urology Centre, Department of Surgery, The Chinese University of Hong Kong, Hong Kong 999077, China; ryantse@surgery.cuhk.edu.hk (R.T.-H.T.); christinewong@surgery.cuhk.edu.hk (C.Y.-P.W.)

**Keywords:** spermine, diacetylspermine, cancer development, polyamines

## Abstract

Polyamines are essential biomolecules for normal cellular metabolism in humans. The roles of polyamines in cancer development have been widely discussed in recent years. Among all, spermine alongside with its acetylated derivative, N^1^, N^12^-Diacetylspermine, demonstrate a relationship with the diagnosis and staging of various cancers, including lung, breast, liver, colorectal and urogenital. Numerous studies have reported the level of spermine in different body fluids and organ tissues in patients with different types of cancers. Currently, the role and the underlying mechanisms of spermine in cancer development and progression are still under investigation. This review summarized the roles of spermine in cancer development and as a diagnostic, prognostic and therapeutic tool in various cancers.

## 1. Introduction

Polyamines are small biomolecules that exist in all organisms and are crucial in many biological functions, ranging from cell growth, gene regulation, and nucleic acid stabilization to cell proliferation [1,2,3]. Nonetheless, intracellular polyamine contents are also found to be greater in tissues or cells with high proliferation rates, for example, cancer cells, as a result of elevated polyamine biosynthesis and uptake [4]. Numerous studies’ verdicts on polyamine content in malignant conditions have been enriched to a large extent [5,6].

Among all, spermine, which is considered one of the most important polyamines produced in mammalian cells were reported to be involved in playing important roles in cell physiology, for instance, ion channel regulation, bone development, inhibition of lipid formation and involvement in the maturation of gut and immune systems [7]. Several studies have attempted to evaluate the normal urinary and serum spermine level. Tsoi et al. determined that the urinary spermine level from normal controls, normalized with creatinine, ranged from 0.63–11.36 μmol/g creatinine, while Durie et al. reported a level of 0.10–1.48 μmol/g creatinine [8,9]. On the other hand, Liu et al. determined a normal urine spermine level of 136.0 ± 410.2 ng/mL [10]. Regarding the spermine level in circulating system, the normal serum spermine levels were ranged from 10–60 ng/mL with a mean of 28 ng/mL and 8.67 ± 7.33 ng/mL as reported from two studies, respectively [10,11]. On the other hand, gender is also a determining factor in polyamine profiles. In males, both urinary and serum spermine levels are significantly higher than those in females (*p* < 0.01) [12].

**Figure 1:**
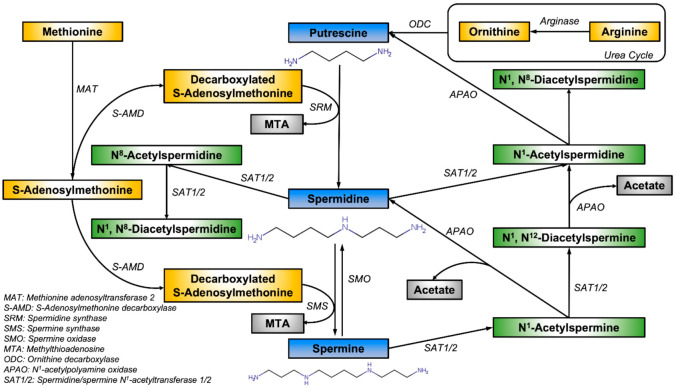
The biosynthesis pathway of spermine and N^1^, N^12^-Diacetylspermine. Spermine biosynthesis starts with decarboxylation of ornithine, catalyzed by ODC, which gives putrescine. Putrescine is catalytically derived into spermidine by SRM and further aminopropylated into spermine, catalyzed by SMS and involvement of SAM. Spermine undergoes oxidation and is derived back into spermidine, which is catalyzed by SMO. Acetylation of spermine, catalyzed by SAT1/2, gives N1-Acetylspermine. Additional acetylation of N1-Acetylspermine produces N^1^, N^12^-Diacetylspermine.

Spermine is synthesized from another polyamine precursor, ornithine. Ornithine was first decarboxylated to putrescine (Put) by ornithine decarboxylase 1 (*ODC*), followed by successive enzyme-catalyzed aminopropyl transfer reactions by spermidine synthase (*SRM*) and spermine synthase (*SMS*) to generate spermidine (Spd) and spermine (Spm), respectively. Conversely, spermine can be catabolized back to spermidine and putrescine by a combination of catabolism involving spermine/spermidine N^1^-Acetyltransferase 1/2 (*SAT1/2*), polyamine oxidase (*APAO*) and spermine oxidase (*SMOX)* (Figure 1) [1].

Nonetheless, spermine, as well as its derivatives, has been shown to be involved in various human cancers’ development. Numerous studies have indicated that the level of spermine, its derivatives, and the enzymes involved in spermine metabolism and catabolism pathways are associated with different malignancies [13]. One of the well explored spermine derivative in cancer is N^1^, N^12^-Diacetylspermine (DiAcSpm), which is produced by two rounds of acetylation of spermine, catalyzed by *SAT1/2*. Studies have demonstrated the prognostic value of DiAcSpm in lung cancer, colorectal cancer and breast cancer [14,15,16]. In this review, we are going to review the changes in spermine level in different human cancers and the possible applications of spermine in cancer treatment regimes in a clinical setup.

## 2. Roles of Spermine in Cancers

The profiles of spermine and acetylated spermine in different malignancies are significantly altered, suggesting that carcinogenesis and disease progression may be the culprit of such changes (Table 1).

### 2.1. Lung Cancer

Since 1978, studies have investigated spermine concentration in different body components in different diseases, including lung cancer and leukemia [29]. Spermine in fingernails was expected to obtain physiologic information and serve as noninvasive samples for disease diagnosis. By using a highly sensitive target-specific derivatization method, the nails of 17 lung cancer patients were compared to 39 healthy controls, and a significant increase of spermine level in their nails were noticed [17,30]. Xu et al. investigated concentrations of a panel of 14 polyamines in the urine and plasma from lung cancer patients as well as healthy volunteers by the LC-Tandem MS method (LC-MS/MS). The authors observed that spermine was the second most abundant polyamine in both urine and plasma of cancerous patients. Moreover, urinary spermine level, but not plasma, was significantly increased in lung cancer patients as compared to healthy individuals [18].

In a recent study investigating the possible role of DiAcSpm as a prognostic non-invasive marker for non-small-cell lung cancer (NSCLC), Takahashi et al. demonstrated that the urinary DiAcSpm levels of healthy controls were significantly lower than that of NSCLC patients, which were in line with the results illustrated by Xu et al. DiAcSpm was useful in distinguishing squamous cell carcinoma from adenocarcinoma and other benign lung diseases, as its level was significantly higher in the former group. Further, the authors also showed that DiAcSpm was reliable in classifying NSCLC patients into different pathological stages. Their results indicated that stage IV urinary DiAcSpm concentration was higher than stages I to III, though not all results reached significance [19].

### 2.2. Liver Cancer

The relationship between spermine concentration and liver cancer development has also been studied by Xu et al. Spermine was found to be the most and third-most abundant polyamine in urine and plasma of cancer patients, respectively, though only urine samples reached a significant level [18].

The clinical significance of DiAcSpm was also evaluated in hepatocellular carcinoma (HCC). It was observed that urinary DiAcSpm levels could distinguish advanced HCC from early-stage HCC and other non-malignant liver diseases. However, the polyamine was incapable of discriminating between early-stage HCC from benign liver diseases, for example, and viral hepatitis and fatty liver patients [20]. Further study on the changes in DiAcSpm levels with treatment was performed by the same group, which showed urinary DiAcSpm levels were significantly reduced in patients received treatment. On the other hand, for five patients who did not undergo any modality of treatments, their urinary DiAcSpm levels were increased in 8 weeks [20]. These suggested the potential role of urinary DiAcSpm levels for treatment monitoring in liver cancer patients.

### 2.3. Breast Cancer

Studies have shown that both serum polyamine profile and spermine/putrescine ratio were significantly increased in breast cancer patients as compared with non-malignant familial polyposis. On the other hand, erythrocyte spermine level and spermine/putrescine ratios showed a significant decrease after surgery and chemotherapy. These suggested the clinical relevance of spermine and its potential role as a tumour marker in breast cancer [21].

DiAcSpm was also investigated by Fahrmann et al. in triple negative breast cancer (TNBC) patients. Its serum level was significantly elevated in cancerous serum when compared to cancer-free controls. The authors also examined the ability to discriminate TNBC from other subtypes of breast cancers and showed promising results. TNBC patients’ serum exhibited a higher DiAcSpm level than non-TNBC and healthy controls. In their prospective cohort, it was observed that serum DiAcSpm level was significantly elevated in patients with early recurrence (<1 year) and was associated with worse 5-year distant metastasis-free survival and 5-year overall survival [15].

### 2.4. Colorectal Cancer

Spermine and its acetylated form are also shown to be promising biomarkers in colorectal cancer (CRC), due to the differential concentrations of urinary expressed polyamines in different clinical conditions. Venäläinen et al. has investigated urinary spermine and DiAcSpm levels in healthy, inflammatory bowel diseases and CRC by LC-MS/MS. DiAcSpm alone performed the best in discriminating CRC with healthy controls with its level significantly elevated in cancerous patients’ urines. In regard to distinguish CRC from benign diseases, urinary spermine was able to distinguish CRC from adenoma, with a diminished urinary level in CRC patients, while the level of urinary DiAcSpm was evaluated in CRC than adenoma. The inconsistence in aberrations demonstrated that a composite model is required for precisive predictions. To correlate polyamine levels with treatment outcomes, unadjusted DiAcSpm level exhibited a significant decreased after a curative operation, but the effect was not observed when its level was normalized with creatinine [22]. Altogether, DiAcSpm might be a potential biomarker in CRC prognosis, while spermine was promising in differentiating malignancies from benign diseases. However, further studies are needed to validate these initial observations.

### 2.5. Urological Cancers

The prostate contains the largest amount of spermine in the body [31] and the spermine levels may alter due to different conditions; for example, benign prostatic hyperplasia (BPH) and prostate cancer (PCa). Tsoi et al. employed ultra-high performance liquid chromatography coupled with a triple quadrupole mass spectrometer to determine the levels of urinary putrescine, spermidine and spermine in cancerous, BPH and healthy conditions. Results indicated that normalized urinary spermine level was significantly lower in cancerous conditions than both BPH and healthy conditions [9]. Similar study with HPLC technique also revealed spermine levels in PCa patients were lowered by 7–34 times in comparison with healthy men and lower by 5–13 times than in patients with BPH as well as in peripheral blood [23]. Serkova et al. has also assessed the role of polyamines in human expressed prostatic secretions by proton nuclear magnetic resonance spectroscopy. The results were quite similar to urinary studies, as regardless of age, spermine concentrations were significantly lowered in PCa subjects [24]. The prostate tissue level of spermine was also found to be reduced in PCa patients, when compared to normal adjacent samples [25,32,33]. Giskeødegård et al. further observed that the concentration of spermine was significantly different between low grade and high grade PCa, with a lower level found higher grade cancers (Gleason score of 8–10) than Gleason 6 cancers [25]. In all these studies, a decrease in spermine concentration was observed in PCa patients, suggesting spermine may be an endogenous inhibitor of PCa growth. The underlying mechanisms led to decrease in spermine level in PCa patients was still uncertainly. Huang et al. have investigated the change in expression levels of *SAT1/2*, an enzyme converting spermine into other metabolites, in human prostate tissue. They observed that *SAT1/2* mRNA levels in epithelial cells were significantly higher in PCa when compared to healthy prostate; however, no significant difference was observed when comparing PCa and BPH as well as localized and aggressive PCa. Nonetheless, metastatic PCa group had a *SAT1/2* mRNA level significantly higher than all other groups. *SAT1/2* protein expression in cellular cytoplasm of prostatic epithelial cells were also investigated. Intriguingly, *SAT1/2* expression was significantly increased in PCa tissues when compared to benign and BPH tissues and was further enhanced in aggressive and metastatic PCa as compared to localized tumors. Surprisingly, subjects with both radiographic and biochemical recurrence expressed a marginally significant increase of *SAT1/2* level in prostate tissue than those without recurrence [34]. Though the change in *SAT1/2* levels could compromise different cellular responses, for instance polyamine oxidation [34,35], it served as potential mechanism for the decrease in spermine concentrations in body fluids and tissues, as well as a diagnostic biomarker for prostate cancer [36].

Few studies in recent years have also investigated the correlation of spermine in urinary bladder cancer and renal cell carcinoma. Spermine profiling in kidney cancer was done as early as in 1970s. Dunzendorfer et al. observed that spermidine levels and spermine concentrations in renal cell carcinoma tissue were elevated when compared to healthy kidney tissues [26]. The accumulation of spermidine in kidney cancer indicated that spermidine turnover rates may be influenced by carcinogenesis, or vice versa. For urinary bladder cancer (BCa), Stejskal et al. attempted to evaluate the usage of DiAcSpm as a potential tumor marker, yet results of urinary level could not distinguish individuals with BCa and controls [27].

### 2.6. Gynaecological Cancers

Progress was also made regarding the alteration of polyamines in ovarian cancers. Niemi et al. investigated 12 polyamines, including spermine and its acetylated forms, in postmenopausal women with ovarian cancers, benign tumors and healthy controls. They observed that six polyamines were constantly found in all urine samples, including DiAcSpm, but not spermine. Nonetheless, DiAcSpm was the only one that showed significance differences, i.e., increased, between cancers and non-cancer patients [28]. Moreover, DiAcSpm showed the ability to discriminate stage III–IV tumors from stage I–II tumors, and high-grade tumors from low malignant potential tumors, therefore exhibiting a positive correlation between urinary DiAcSpm concentration and clinical stages as well as histological grades [28].

## 3. Applications and Prospects

In recent years, polyamines have gained interest by translating laboratorial results into novel therapeutic agents. With advanced technology, precise levels of biomolecules are able to be detected in different body fluids, and significant aberrations have been observed in various conditions. Therefore, polyamines, such as spermines and DiAcSpm, could be applied as potential biomarkers for diagnosis and prediction of clinical outcomes, and also as potential new therapeutic target for malignancies.

### 3.1. Detection and Prognosis

Although spermine and its acetylated forms were shown to alter in different human cancers, comparison with conventional clinical screening tests should be performed before serving as a detection alternative in clinical setting. Tsoi et al. attempted to compare conventional serum prostate-specific antigen (PSA) with urinary spermine as a second screening test in prostate cancer. They demonstrated that urinary spermine achieved a sensitivity and specificity of 67.05% and 68.75%, respectively, with an outstanding screening performance (AUC = 0.83 ± 0.03) focusing on patients with PSA > 4.0 ng/mL [9]. With regards to current serum, a PSA test only yielded a poor to fair screening power accompanied with over-diagnosis and unnecessary prostate biopsies [9,37]. Further prospective study conducted by Chiu et al. not only confirmed urinary spermine could help to decrease unnecessary prostate biopsy due to elevated serum PSA, but also that a decreasing level of urinary spermine was associated with progressive increase prostate cancer grade [38].

Sensitivity and specificity of DiAcSpm was also shown to be comparable with well-known serum tumor markers for HCC. Enjoji et al. reported the sensitivity of DiAcSpm (65.5%) was neither superior nor inferior to alpha-fetoprotein (AFP) (63.8%) and protein induced by vitamin K absence or antagonist II (PIVKA-II) (60.3%), illustrating that the polyamine, either alone or in conjunction with AFP and PIVKA-II, could be a reliable non-invasive index in HCC detection, especially in patients without elevation of other serum markers [20]. Moreover, DiAcSpm could also serve as a potential candidate in ovarian cancer screening tests. Screening performance of DiAcSpm was compared to serum cancer antigen 125 (CA125) markers. DiAcSpm showed a better sensitivity, despite lower specificity in distinguishing malignant tumors from benign tumors (AUC = 0.83) than CA125 (AUC = 0.81) [28].

DiAcSpm was also investigated as a pre-diagnostic serum marker in NSCLC. Wikoff et al. compared the performance of the polyamine with pro-surfactant protein B (pro-SFTPB), a previously established protein biomarker for NSCLC. The authors demonstrated that DiAcSpm yielded an AUC of 0.657, which was significantly complemented the performance of pro-SFTPB (AUC = 0.635). Further, results indicated that DiAcSpm levels were significantly higher in serum samples collected closer to the timepoint before diagnosis, suggesting the association of increased serum DiAcSpm levels with tumor development and progression. The authors also observed an additive performance between DiAcSpm and pro-SFTPB, which yielded an overall AUC of 0.732 and an AUC of 0.808 in serum samples 0 to 6 months before diagnosis [16].

Spermine was also utilized to detect and discriminate pancreatic cancer from other malignancies. Salivary spermine level in pancreatic cancer patients was a potential indicator of pancreatic cancer as spermine exhibited a specific and increased level in cancerous patients [39]. On the other hand, unlike traditional blood-taking-based cancer screening tests, being a non-invasive liquid biopsy, urine and saliva can be readily and easily obtained from patients without causing any discomfort. Nonetheless, volume and period of specimen collection should be standardized.

### 3.2. Novel Therapeutic Agents

Since spermine and DiAcSpm levels are altered in different cancers, this suggests that the polyamines themselves can be targets of novel therapies. In lung cancer, the role of spermine in the A549 lung cancer cell line was investigated. Selenomethionine could induce apoptosis in A549. With the addition of 65 μM of selenomethionine for 24 h, the level of spermine was greatly decreased, while the level of spermidine was only slightly depleted. Redman et al. also concluded that spermine could protect cancer cells from the growth-inhibitory effects induced by selenomethionine as the percentage of apoptotic cells after the addition of exogenous spermine returned to control levels. This suggested that selenomethionine could be a hypothetic treatment targeting spermine and it was believed that suppression of spermine in A549 leaded to induction in apoptosis and perturbations in cell cycle [40]. Moreover, previously published studies had suggested that oxidative stress expansion could lead to lung cancer and tried to establish a novel treatment modality.

Atorvastatin has also been applied in Benzo(a)pyrene (BaP)-induced lung cancer rats to modulate polyamines level and as a treatment strategy of lung cancer. Atorvastatin was found to downregulate polyamine levels, such as histamine, spermine, spermidine and putrescine, significantly (*p* < 0.001), leading to a reduction in pro-inflammatory cytokines such as caspase-3, Bax levels, whereas upregulation in Bcl-2 level to regulate apoptosis [41]. Moreover, other common targets of spermine in lung cancer could also be used as antineoplastic therapies. Several spermine congeners, such as Bisethylspermine (BESpm), a symmetrically substituted spermine analogue, were found to have an unusual property to increase the activity of polyamine catabolic enzyme *SAT1/2* which resulted in the depletion of intracellular polyamines, cell growth inhibition in a tumor-specific manner and induced programmed cell death by apoptosis [42,43]. These observations suggested that spermine and its related molecules could be potential candidates in treating lung cancers.

A functional study investigated the effects of spermine on estrogen receptor ER DNA-binding (ER-ERE complex), ER ligand-binding (estradiol), ER structure (circular dichroism and sucrose gradient sedimentation) and the capacity of ER to transactivate ERE-tk-CAT reporter in ER-positive MCF-7 and ER-negative MDA-453 breast cancer cell lines had been performed [44]. Results showed that 3 mM spermine caused 50% inhibition of ER-ERE formation, <0.1 mM spermine could inhibit ER ligand-binding, which correlated with higher-order ER structure and suppression of intracellular ER transactivation [44]. These suggested that spermine was an important polyamine in regulating the ligand-binding and gene-activating functions of ER; this might provide hints towards the reasons underlying ER-positive breast tumors that escaped from endocrine therapy. In recent years, other aspects of spermine in breast cancer had also been proposed. The effects of extracellular spermines on the breast cancer cell line BT474 were tested, which showed that spermine elicited a Ca^2+^ signaling composed of both Ca^2+^ release and Ca^2+^ influx and could decrease cell line viability by apoptosis. This suggested spermine cytotoxicity could act through another in vitro mechanism, i.e., acting on a putative polyamine receptor in BT474 cells instead of oxidative stress, resulted in Ca^2+^ overload, Ca^2+^ store depletion and ER stress [45]. The results indicated that spermine can be employed as a polyamine target in tackling cancers; nonetheless, intensive experiments on cell lines, xenograft models and clinical trials are required before applying results to clinical atmosphere.

The pathway of spermine metabolism was also utilized as a target in cancer therapy. Difluoromethylornithine (DFMO) was widely studied as an anticancer and chemoprevention agent. DFMO is an irreversible suicide inhibitor of *ODC*, which is responsible in the decarboxylation of ornithine into putrescine, which is subsequently derived into spermidine and spermine. DFMO exhibits its inhibitory effect by enzymatic decarboxylation and binds with active site of *ODC* and blocking the transcription of c-myc [46]. Due to its chemopreventive effects, DFMO was adopted in clinical trials to thwart various cancer. For example, DFMO was investigated in CRC for its anticancer effects. Studies indicated that DFMO inhibited colon carcinogenesis in xenograft models, including *Apc*^Min/+^ mice, suppressing carcinogenesis in the small and large intestine of xenografts, both alone and synergized with non-steroidal anti-inflammatory drugs (NSAIDs), especially sulindac, as indicated from recent clinical trials [47]. Nonetheless, cancer cells can overcome *ODC* depletion by importing polyamines from extracellular sources, compensating the treatment effects of DFMO. Therefore, recent studies demonstrated that dual administration of a novel polyamine transporter inhibitor AMTX-1501 and DFMO was a potent combination in significantly depleting polyamine levels, including putrescine, spermidine and spermine. Followed by a reduction in cell proliferation, clonogenic potential, cell migration and induction of apoptosis both in vitro and in vivo in diffuse intrinsic pontine glioma (DIPG), survival in orthotopic models of DIPG was also enhanced [48]. As a result, DFMO, alone or combining with drugs and polyamine transporter inhibitors, not only inhibited production of polyamines, but also prevented the uptake of extracellular polyamines, such as spermidine and spermine, to avoid accelerating the growth of established tumor [49].

Photodynamic therapy (PDT) is another treatment modality in cancer therapies involving the intravenous, intraperitoneal or subcutaneous injection of photosensitizer (PS) and allows direct tumor elimination, microvascular damage, tumor hypoxia and stimulation of immune response after treatment. Nonetheless, commercially available photosensitizers displayed significant drawbacks, such as toxicity, low penetration ability, low blood solubility and low tumor selectivity. Recently, a study demonstrated spermine could be conjugated with photosensitizers to enhance phototoxicity to cancer cells in vitro. Darmostuk et al. attempted to develop conjugate spermine and purpurin 18 or pheophorbide a. Results showed that spermine/PS conjugate did not cause toxicity to non-cancerous cell lines, but induced strong phototoxicity towards HeLa, MCF7 and LNCaP cell lines, representing cervical, breast and prostate cancers, respectively. In addition, spermine/PS conjugate exhibited superior retention in cancer cells over PS alone. The additional advantage of spermine/PS conjugate, which possessed high phototoxic activity permitted its usage in low concentration, may allow it to overcome a common disadvantage of PDT, i.e., potential necrotic damage under a high concentration of photosensitizers. This is supported by a study using a low concentration of spermine/PS that resulted in apoptosis, in a PS concentration and light dose-dependent manner, as shown by flow cytometric results [50]. The study result revealed that the spermine/PS conjugate could serve as a basis of next-generation PS in vivo and the value of spermine being employed in establishing novel therapeutic agents.

## 4. Conclusions

Variable polyamine profiles have been shown in various human cancers and benign diseases. Among all reported polyamines, spermine and DiAcSpm were shown to have strong clinical relevance. Aberrations of spermine and DiAcSpm levels in different body fluids, such as urine, plasma and tissue are reported in this review. Numerous studies have demonstrated that spermine and DiAcSpm can discriminate cancerous patients from healthy individuals. Additionally, in some cancers, spermine and DiAcSpm are able to differentiate malignancies according to different clinical stages and histological grades, demonstrating their sensitivity and specificity in cancer research. Moreover, spermine and its related molecules can also be employed in developing therapeutic agents. An increasing number of studies have attempted to utilize the polyamine as a target to inhibit cell progression and development, as well as coupling with commercially available drugs. Nonetheless, more laboratorial works in vitro and validations on xenograft models are required in the future before translating results into clinical treatments.

## Figures and Tables

**Table 1:** Summative table of the change in spermine and diacetylspermine concentrations in different human cancers.

Cancer of Origin	Method	Major Outcome	Polyamine Levels ^	Statistical Significance	AUC	Reference
Lung	LC-ESI-MS/MS	Increased fingernail Spm level	NR	*p* < 0.05 ^a^	NR	[17]
	UHPLC-MS/MS	Increased urinary Spm level	0.30 ± 0.36 ^‡^; 0.80 ± 0.86 ^‡^; 2.67	*p* < 0.01 ^a^	NR	[18]
	UHPLC-MS/MS	Decreased plasma Spm level	12.61 ± 12.02 ^†^; 6.78 ± 3.87 ^†^; 0.54	*p* > 0.05 ^a^	NR	
	Colloidal gold aggregation	Increased urinary DiAcSpm level in NSCLC	0.0005 (0.485–0.847) ^‡^; 0.0008 (0.605–1.28) ^‡^; 1.60	*p* < 2.2 × 10^−6 a^	0.75	[19]
Liver	UHPLC-MS/MS	Increased urinary Spm level	0.30 ± 0.36 ^‡^; 1.88 ± 2.34 ^‡^; 6.27	*p* < 0.01 ^a^	NR	[18]
	UHPLC-MS/MS	Increased plasma Spm level	12.61 ± 12.02 ^†^; 14.24 ± 10.73 ^†^; 0.89	*p* > 0.05 ^a^	NR	
	ELISA	Increased urinary DiAcSpm level in advanced HCC	Threshold set at 325 nM/g creatinine	*p* < 0.0001 ^a,b^	NR	[20]
Breast	NR	Increased serum Spm level and Spm/Put ratio	NR	NR ^b^	NR	[21]
	LC-MS	Increased plasma DiAcSpm level in TNBC	0.98 ± 0.05; 1.11 ± 0.30; 1.13	*p* < 0.001 ^a^	0.64	[15]
Colorectal	LC-MS/MS	Increased urinary DiAcSpm level	0.0855 (0.15) ^‡^; 0.182 (0.20) ^‡^; 2.13	*p* = 0.00049 ^a,b^ *p* = 0.042 ^b^	0.72	[22]
Prostate	UHPLC-MS/MS	Decreased urinary Spm level	5.43 ± 1.17; 1.47 ± 0.22; 0.27	*p* < 0.0001 ^a,b^	0.83 ± 0.03	[9]
	HPLC	Decreased urinary Spm level	NR	NR	NR	[23]
	^1^H-NMRS	Decreased prostatic secretion Spm level	NR	*p* < 0.002 ^a^	0.79	[24]
	HR-MAS	Decreased tissue Spm level in PCa	1.92 (0.86–3.13) ^#^; 1.22 (0.66–2.00) ^#^; 0.635	*p* = 0.022 ^a^	0.86 *	[25]
Kidney	Amino acid analyzer	Decreased tissue Spm level	6.85 ± 6.97; 4.05 ± 2.23; 0.59	*p* = 0.1 ^a^	NR	[26]
Urinary Bladder	ELISA	No significant urinary DiAcSpm level change	NR	*p* = 0.64 ^a^	<0.7	[27]
Ovary	LC-MS/MS	Increased urinary DiAcSpm level	0.10 ± 0.04 ^‡^; 0.54 ± 0.62 ^‡^; 5.4	*p* < 0.001 ^a,b^	0.83	[28]

Abbreviations: LC-ES-MS/MS, liquid chromatography-electrospray ionization-mass spectrometry; UHPLC-MS/MS, ultra-high performance liquid chromatography tandem mass spectrometry; ELISA, enzyme-linked immunosorbent assay; 1H-NMRS, proton nuclear magnetic resonance spectrum; HR-MAS, high-resolution magic angle spinning; Spm, spermine; DiAcSpm, N1, N12-diacetylspermine; Put, putrescine; NSCLC, non-small-cell lung carcinoma; HCC, hepatocellular carcinoma; TNBC, triple negative breast cancer; AUC, area under the ROC curve; NR, not reported. ^ Polyamine levels were represented in the order of benign; cancer; fold change (cancer level/benign level). ^‡^ Polyamine levels were represented in mean ± SD μmol/g creatinine or median (IQR) μmol/g creatinine. ^†^ Polyamine levels were represented in mean ± SD ng/mL. ^#^ Polyamine levels were represented in mean ± SD mmol/kg tissue samples or median (IQR) mmol/kg tissue samples. ^a^ Polyamine levels compared with healthy controls. ^b^ Polyamine levels compared with benign disease individuals or healthy controls. * Composited model included spermine.

## Data Availability

All available data is presented in the manuscript.
